# Clinical application of ^192^Ir High-Dose-Rate brachytherapy in metastatic lymph nodes of the neck

**DOI:** 10.1007/s12672-023-00827-8

**Published:** 2023-12-01

**Authors:** Hongling Lu, Yunchuan Sun, Yan Gao, Li Xiao, Jianxi Zhou, Xiaoming Yin, Wei Guo, Kui Fan

**Affiliations:** Department of Radiation Oncology, Cangzhou Hospital of Integrated Traditional Chinese and Western Medicine-Hebei Province, No. 31, Huanghe West Road, Yunhe District, Cangzhou, 061000 Hebei China

**Keywords:** High-dose-rate brachytherapy, Secondary malignancy of lymph nodes in the neck, Local control rates, Adverse effects

## Abstract

**Objective:**

The objective of this study was to investigate the safety and effectiveness of high-dose-rate brachytherapy as a treatment modality for recurrent or residual neck metastatic lymph nodes following external radiotherapy.

**Methods:**

38 patients with 52 metastatic lymph nodes recurring or residual after previous external radiotherapy was completed to metastatic lymph nodes in the neck were collected from January 2019 to February 2022. High-dose-rate brachytherapy with ^192^Ir was performed with a prescribed dose of 20–30 Gy/1f (effective biological dose of 60–120 Gy), and imaging was performed at 1, 3, and 6 months after treatment to assess the local control rate and adverse effects of treatment.

**Results:**

All 38 patients received completed treatment, and they were followed up for 6 months. 52 patients with neck lymph node metastases had an objective response rate.

(Complete response, CR + Partial response, PR) of 76.9%, which comprised 89.5% (34/38) for lymph nodes ≤ 3 cm and 42.9% (4/14) for > 3 cm, *P* = 0.028. *P* > 0.05 for CR + PR versus stable disease, SD + progressive disease, PD for lymph nodes between different subdivisions of the neck. Using the Radiation Therapy Oncology Group (RTOG) Acute Toxicity Scoring System, there were 6 cases of acute radioskin injuries of degree I and 4 cases of degree II with a 60% symptomatic relief rate.

**Conclusions:**

High-dose-rate brachytherapy serves as a safe and effective method in treating recurrent residual neck metastatic lymph nodes in the field after external radiotherapy, exerting tolerable adverse effects.

## Introduction

Neck lymph nodes have been confirmed as the most frequent sites of lymphatic metastasis from head, neck and chest malignancies. In general, patients subjected to neck lymph node metastasis suffer from pain and compression of essential organs (e.g., blood vessels and nerves), leading to the significantly decreased quality of life. Notably, for recurrent or residual metastatic lymph nodes after external radiotherapy, the efficacy of systemic treatment is often poor for factors (e.g., failure to achieve local drug concentration in tissue fibrosis), and local treatment is usually required (e.g., lymph node dissection, recourse external radiotherapy, particle implantation, as well as high-dose-rate brachytherapy). With the continuous improvement of radiotherapy technology, high-dose-rate brachytherapy has been widely used. Collettini et al. [1] have suggested that high-dose-rate brachytherapy is a safe and effective technique for minimally invasive ablation of lymph node metastases. In this study, recurrent or residual metastatic lymph nodes were administrated with ^192^Ir high-dose-rate brachytherapy in the neck after external radiotherapy. A retrospective analysis of 38 patients was conducted to assess the safety and efficacy of ^192^Ir high-dose-rate brachytherapy for recurrent or residual metastatic lymph nodes in the neck after external radiotherapy.

## Materials and methods

### Patients

38 tumor patients with metastatic lymph nodes in the neck after external radiotherapy from January 2019 to February 2022 were collected. Twenty-one of the 38 patients in this study had definitive pathology by fine-needle aspiration biopsy, and the remaining 17 were clinically diagnosed by imaging (6 by PET-CT, 4 by Magnetic Resonance Imaging, and 7 by Contrast-Enhanced CT). Surgery was not recommended after head and neck surgery consultation or the patient refused surgery. At the same time, this treatment protocol complied with ethical standards for human trials and the Declaration of Helsinki, and informed consent was obtained from the patient. Among them: 16 males and 22 females; median age 65 (53–77) years; Eastern Cooperative Oncology Group(ECOG) performance status score 0 in 6 patients, score 1 in 22 patients, and score 2 in 10 patients. Among them were 12 patients of nasopharyngeal cancer, 4 patients of hypopharyngeal cancer, 2 patient of tonsil cancer, 6 patients of esophageal cancer, 6 patients of breast cancer, and 8 patients of lung cancer.

Four of the twelve patients with nasopharyngeal carcinoma were administrated with targeted chemotherapy followed by combined radiotherapy and eight with simultaneous radiotherapy at doses (PGTV (The planning gross tumor volume) = 68–72 Gy; PGTVnd (The planning gross target volume of metastatic lymph nod) = 66 Gy; PTV1 (planning target volume) = 54–60 Gy; PTV2 = 50–54 Gy). Four patients with hypopharyngeal cancer were administrated with pharmacological chemotherapy followed by radical radiotherapy with radiotherapy doses (PGTV = 66–69.96 Gy; PGTVnd = 66 Gy; PTV1 = 54–60 Gy; PTV2 = 50–54 Gy). Radical radiotherapy (PGTV = PGTVnd = 66 Gy; PTV1 = 60 Gy; PTV2 = 54 Gy) for patients with tonsil cancer who refused chemotherapy. Four of the esophageal cancer patients were treated with sequential radiotherapy and two with concurrent radiotherapy (PGTV = 60 Gy; PGTVnd = 66 Gy; PTV = 54 Gy). The breast cancers were all triple-negative breast cancers administrated with conventional postoperative radiotherapy (PTV = 50 Gy) and recurrence followed by multiple cycles of chemotherapy. Among the 8 lung cancer cases, 5 adenocarcinoma cases (without gene mutation) were treated with multiple cycles of chemotherapy combined with or without immunotherapy, 2 squamous carcinoma cases were treated with multiple cycles of chemotherapy combined with immunotherapy, and 1 small cell lung cancer case was treated with multiple cycles of chemotherapy and radiotherapy to the chest and neck (PTV = 50–54 Gy; PGTV = 60 Gy; PGTVnd = 60–66 Gy).

Status before high-dose rate brachytherapy: the diagnosis was confirmed through pathology or imaging. The data were acquired from patients with metastatic lymph nodes in the neck. All metastatic lymph nodes were recurred in the field after external radiotherapy. There were 28 residual lymph nodes and 24 new metastatic lymph nodes after radiotherapy and comprehensive treatment, 38 patients had lymph nodes ≤ 3 cm in size, 14 patients had lymph nodes > 3 cm, which comprised 4 patients with lymph nodes > 5 cm and unclear boundaries with surrounding tissues.

## Radiotherapy

**Fig. 1:**
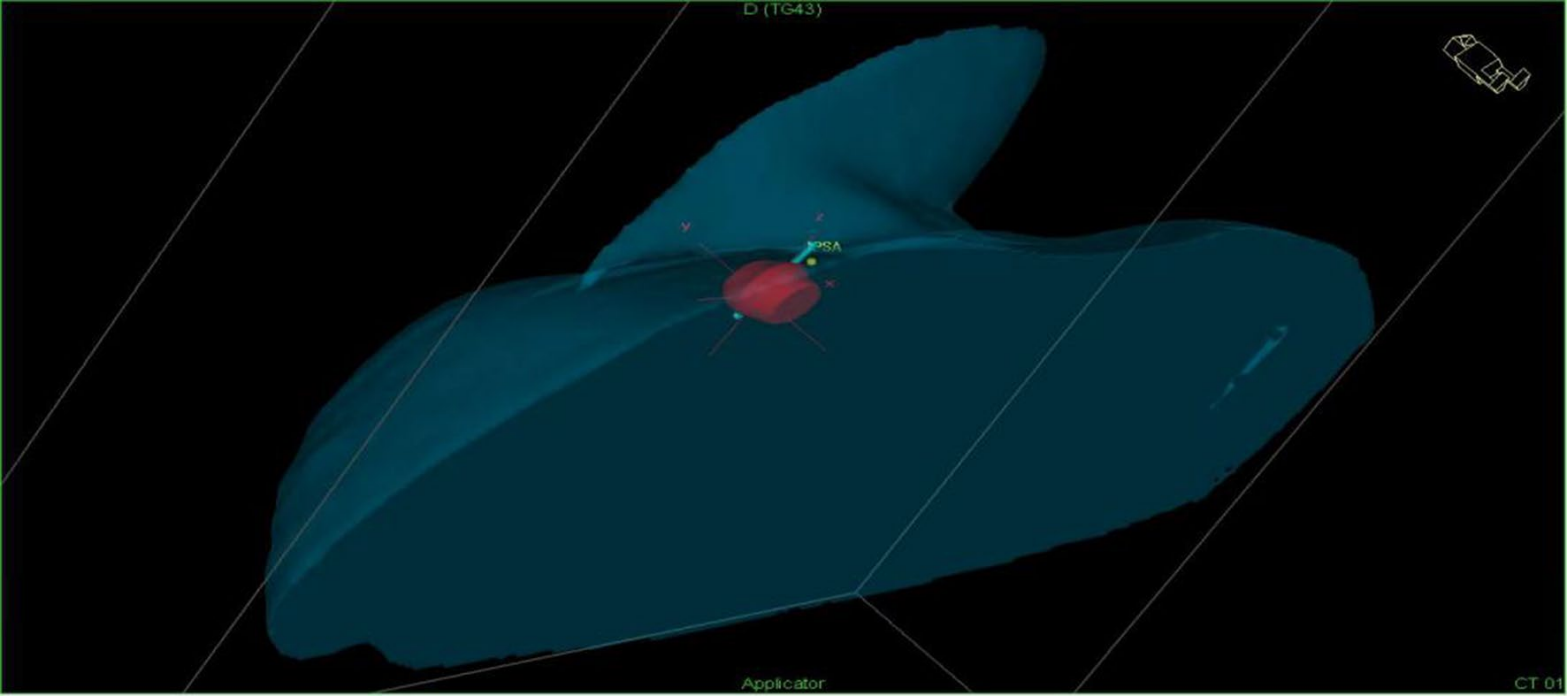
3d reconstruction after implantation of insertion pins

**Fig. 2:**
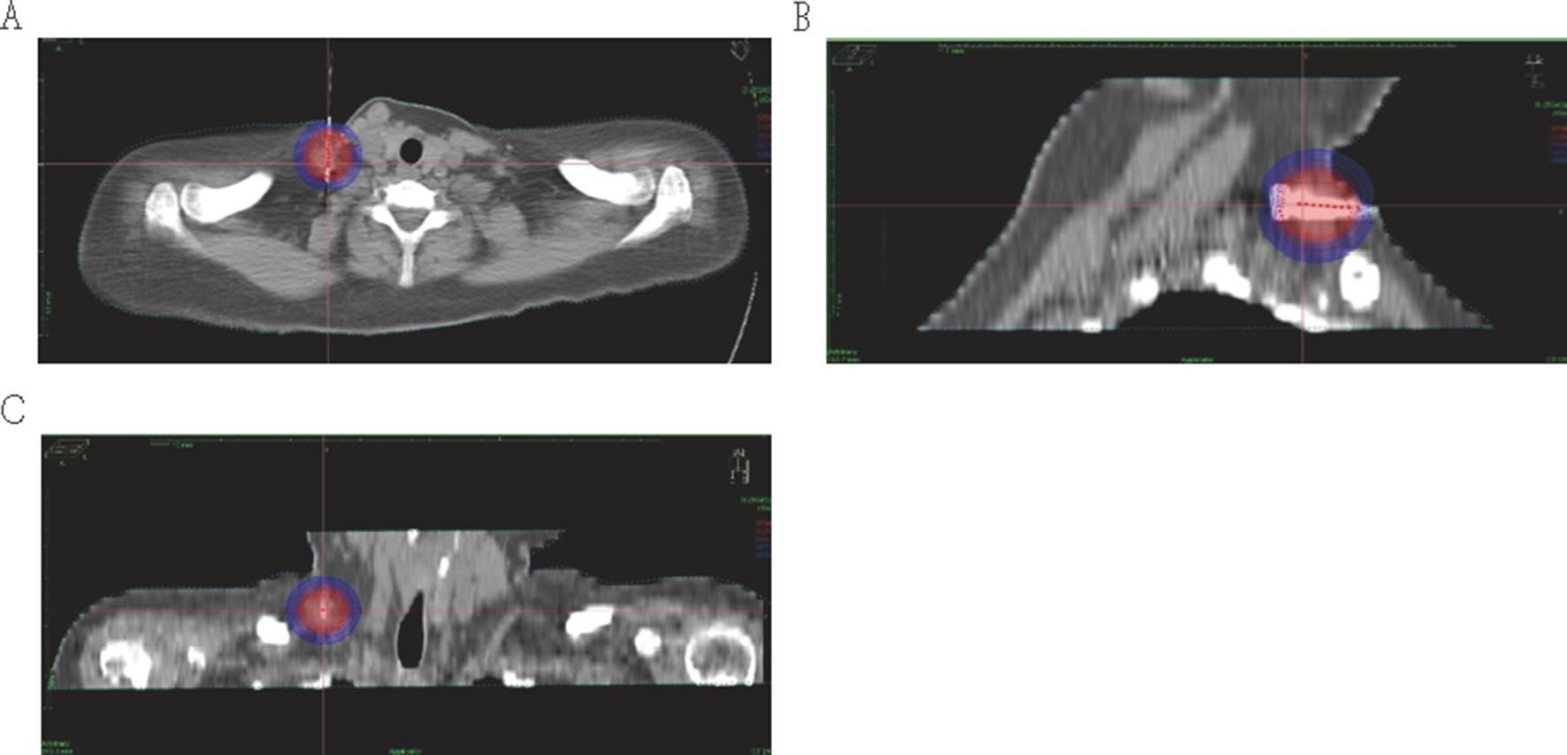
(**A** cross-sectional) Images of a patient with neck lymph node metastases treated with high-dose-rate brachytherapy plan, (**B** sagittal) Image of a patient with neck lymph node metastasis treated with high-dose-rate brachytherapy plan, (**C** coronal) High-dose-rate brachytherapy planning images for patients with neck lymph node metastases

**Fig. 3:**
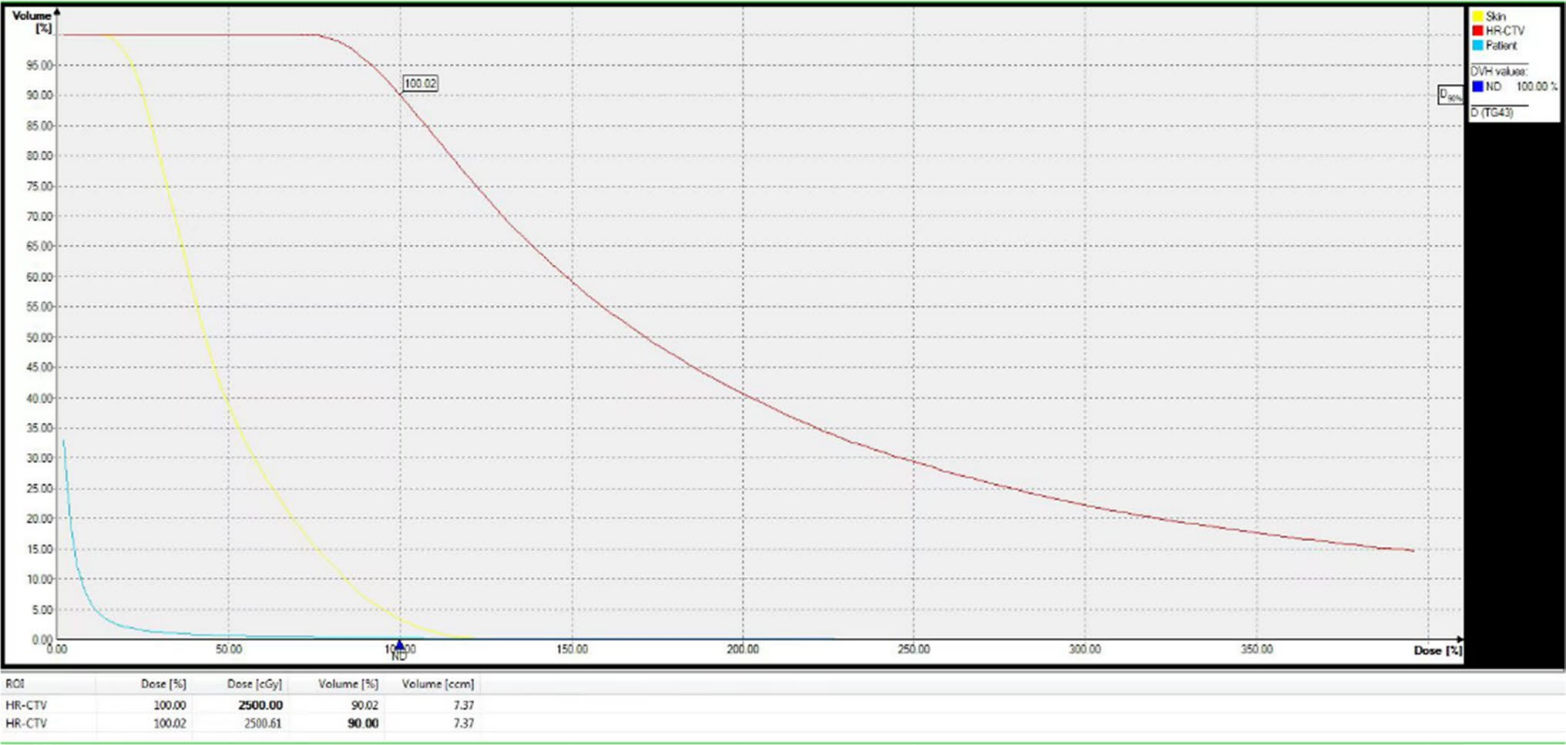
High-dose-rate brachytherapy planning for patients with neck lymph node metastases

(1) Make preoperative plan: the CT images are scanned, the appropriate puncture route is selected in accordance with the location and size of the enlarged lymph nodes and surrounding organs, and a preoperative plan is made. (2) Puncture: lead wire calibration is performed to determine the puncture site, routine disinfection and towel laying, local infiltration anesthesia, implantation of the insertion needle, CT scan to confirm the arrival of the predetermined location, 3D reconstruction is performed (Fig. 1), the location of the puncture needle is clarified, the layer thickness of 3 mm images transmitted to the OncentraBrachy system (Sweden by the company of ELEKTA) is acquired. (3) ^192^Ir high dose rate brachytherapy implementation: the target area and surrounding organs at risk are outlined, a radiotherapy plan is developed (single dose of 20–30 Gy covering 95% of the tumor target area) (Fig. 2a–c), the plan is reviewed by senior physicians (Fig. 3), and the ^192^Ir radiation source for afterloading radiotherapy is connected after the plan is approved (stepwise 2.5 mm source residency spacing in the target area). The puncture needle was removed and pressure bandaged after ^192^Ir high-dose rate brachytherapy was completed, and CT was reviewed to clarify whether there were adverse effects (e.g., bleeding).

## Follow-up and evaluation

**Fig. 4:**
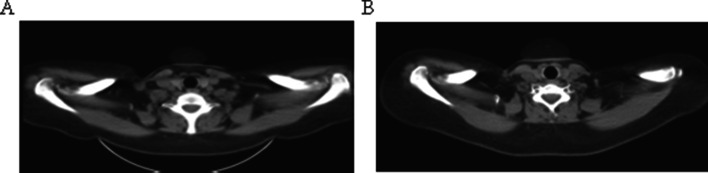
Comparative efficacy evaluation of high-dose rate brachytherapy before and after

Postoperative efficacy was assessed regularly through CT and other imaging examinations (Fig. 4). It was classified in accordance with RECIST1.1 criteria as complete disappearance of tumor lesions (CR), reduction of the sum of the maximum single diameter of tumor lesions by over 30% (PR), increase of the sum of the maximum single diameter of tumor lesions by 20% or appearance of new lesions (PD), reduction of tumor lesions that did not reach the degree of PD increase nor the level of PD (SD), and CR + PR was considered objective response rate. Adverse reactions conformed to the RTOG criteria in terms of acute radiation injury.

## Statistical methods

SPSS 22.0 was employed for statistical analysis. The measurement data are expressed as mean ± standard deviation, the count data are expressed as frequency and composition ratio, and the chi-square test or Fisher's exact probability method was adopted for the comparison between groups. The test level *α* = 0.05, and *P* < 0.05 indicated a difference that achieved statistical significance.

## Results

### Overall treatment of metastatic lymph nodes is effective

At 6-month follow-up, the overall remission rate (CR + PR) of 52 lymph nodes was 76.9%. Among the lymph nodes less than or equal to 3 cm, there were 12 cases of CR, 22 cases of PR, 4 cases of SD, and 0 cases of PD, with an objective remission rate of 89.5% (34/38), and among the lymph nodes greater than 3 cm, there were 2 cases of CR, 4 cases of PR, 4 cases of SD, and 4 cases of PD, with an objective remission rate of 42.9% (6/14), *p* = 0.028. No statistically significant difference was identified between the remission rate (CR + PR) and non-remission rate (SD + PD) of lymph nodes in different subdivisions of the neck (*P* > 0.05) (Table 1). To be specific, 4 patients with enlarged lymph nodes > 5 cm and unclear relationship with surrounding tissues progressed after subjected to high-dose rate post-loading radiotherapy.

**Table 1:** Relationship between lymph node neck levels and efficacy

Lymph node levels of the neck	Number of lymph nodes(n)	CR + PR(n)	SD + PD(n)
Level I	10	8	2
Level II	4	2	2
Level III	14	10	4
Level IV	16	12	4
Level V	8	6	2

*CR* Complete response, *PR* Partial response *SD* Stable disease, *PD* Progressive disease

### Adverse effects can be tolerated

In 4 patients where the skin damage was designated as degree I after radiotherapy, moderate edema progressed to degree II after the insertion of post-implantation mounted radiotherapy; in 6 patients where the skin damage was designated as degree 0 after radiotherapy, dry desquamation progressed to degree I after the insertion of post-implantation mounted radiotherapy. There was no degree III or IV skin damage. Improvement after giving symptomatic treatment. Bone marrow suppression: 2 patients of leukopenia grade I, which improved after giving white-raising treatment. There were no serious complications such as hemorrhage, infection, or other organ damage.

### Improves patients' local symptoms and quality of life

The symptoms before brachytherapy were apparent, including pain in 10 patients, dysphagia in 4patients, facial edema in 6 patients, and no apparent symptoms in the rest. After treatment, 10 patients had pain due to compression of peripheral nerves, among which 8 patients had significant pain relief after brachytherapy; 4 patients had no significant improvement in dysphagia; 6 patients had facial edema, 4 patients had significant relief and one patient had no significant change. The overall symptom relief rate was 60%.

## Discussion

Metastatic lymph nodes often need intervention after comprehensive treatment of head, neck and chest tumors. Since the first course of radiotherapy often leads to local tissue fibrosis and vascular occlusion, it is difficult for drugs to pass through, and coupled with the poor physical condition of patients with advanced tumors, it is difficult to tolerate the adverse reactions arising from chemotherapy and other drugs, local treatment becomes an essential part. Temam et al. [2] have suggested that only 20% of patients with recurrence can undergo palliative surgery due to local structural disturbances arising from the initial surgery and external radiation. With the continuous improvement of radiotherapy techniques, high-dose rate brachytherapy has been well employed in a wide variety of solid tumors [3, 4] (e.g., good results in metastatic lymph nodes) [5, 6]. In this study, 38 patients who received ^192^Ir high-dose-rate brachytherapy for neck metastatic lymph nodes that recurred in the field after radiotherapy were analyzed retrospectively.

The recent efficacy is an important index to assess the treatment effect. The efficacy evaluation results of this study indicated that the overall remission rate (CR + PR) of 52 lymph nodes reached 76.9%, comprising 89.5% (34/38) for lymph nodes less than or equal to 3 cm and 42.9% (6/14) for lymph nodes over 3 cm, *P* = 0.028. In the previous literature, in Nikolaos Tselis et al [7], 74 patients were treated for inoperable recurrent cervical lymphadenopathy with ^192^Ir High-Dose-Rate Brachytherapy, with a 67% probability of local control at 1, 2, and 3 years. In Christos Kolotas et al [8], 49 recurrent metastatic neck lesions underwent ^192^Ir High-Dose-Rate Brachytherapy, with a response rate of 83% (41/49), a complete response of 20% (10/49), a partial response of 63% (31/49), and 8/49 patients (17%) who did not respond to the treatment at a minimum of 6 weeks of follow-up. After a median follow-up of 19 months, the local control rate was 69%, with 15/49 patients (30%) experiencing localized disease progression. Although the overall response rate of this study is similar to the above studies, the follow-up period is still short, and the evaluation of long-term efficacy needs to be further analyzed.

Particle therapy, a minimally invasive treatment modality, has accumulated considerable experience treating neck lymph nodes. In a study by Jiang Yuliang et al. [9], the recent efficacy of ^125^I particles in 36 patients with recurrent neck lymph nodes after radiotherapy for head and neck tumors was manifested as seven patients of complete remissions, 21 patients of partial remissions, six patients of no changes, and two patients of progressions in the whole group, at an effective rate of 81%. The recent efficacy of this study is similar to it. Both modalities are similar in that they can achieve rapid dose drop in a small area, killing the tumor by high dose radiation and protecting the surrounding endangered organs well. Moreover, tumors are often resistant to radiotherapy in terms of patients previously administrated with radiotherapy. Relevant literature has suggested that the radiation-resistant tumor treatment effect can be increased by increasing the single dose, reducing the number of divisions, and shortening the treatment time. Under the effect of the single large dose of radiation energy in high-dose rate brachytherapy, tumor cells become more susceptible to lethal injury, anti-tumor immunity is induced, T-cell immunity is activated, and a regional or distant metastasis distant tumor suppression effect is formed [10, 11]. On the other hand, the correlation between lymph node size and the efficacy of high-dose rate brachytherapy was analyzed in this study. In the study by Wang Juan [12]et al., 17 patients were administrated with recurrence of metastatic lymph nodes in the neck who had previously received external radiotherapy with particle implantation. The local control rate reached 65.2%, where the control rate of lymph nodes smaller than 4 cm (CR + PR) was determined as 90%, while the control rate of lymph nodes larger than 4 cm was 46%, *P* = 0.038; the difference achieved statistical significance; the control rate of 21 lymph nodes with isolated and clear borders was obtained as 71%, and the control rate of lymph nodes with fusion and unclear borders reached 0. The control rate of 21 isolated lymph nodes with clear borders determined as 71%, and the local control rate of fused lymph nodes with unclear borders reached 0. In another study, Ashamalla et al. [13] reported a local control rate of 64% for head and neck malignancies < 2.5 cm in diameter and 33% for > 2.5 cm in diameter using radioactive particles. The present study also showed poor control of larger lymph nodes compared to the above two studies. To be specific, 12 patients of CR, 22 patients of PR, 4 patients of SD, and 0 patients of PD in lymph nodes less than or equal to 3 cm, with an objective remission rate of 89.5% (34/38), and 2 patients of CR, 4 patients of PR, 4 patients of SD, and 4 patients of PD in lymph nodes greater than 3 cm, with an objective remission rate of 42.9% (6/14), *P* = 0.028, which achieved statistical significance, suggesting the effect of high-dose rate brachytherapy can be accurate for ≤ 3 cm lymph nodes. Although no statistical difference was identified between the different subdivisions of the neck and the efficacy in this study, Level II and III of the neck were adjacent to the spinal cord, pharynx, large blood vessels in the neck and other endangered organs, such that the difficulty of needle access was often increased, and it was also limited by the dose. Four patients with unclear relationship with surrounding tissues and enlarged lymph nodes larger than 5 cm were in Level III in this study, two of which was adjacent to the spinal cord and two to the pharynx, and the prescribed dose was 20 Gy/1f in two patients, with an effective peripheral biological dose of 60 Gy and short-term progression after loading at high-dose-rates. There are two reasons for the above-mentioned result. 1. large tumor size. As the tumor size is increased, the blood supply to the center of the tumor becomes poorer, and the percentage of hypoxic cells is increased, resulting in increased radiation resistance. 2. insufficient dose. Fletcher et al. [14] reported that for a 5-cm diameter adenocarcinoma tumor, the external irradiation dose should be 80–90 Gy or even 100 Gy, and the peripheral dose is a direct factor affecting the efficacy. The peripheral dose exerts a direct effect on the efficacy, which may be better if the peripheral dose exceeds 75 Gy. In contrast, the prescribed dose of high-dose rate brachytherapy given to metastatic lymph nodes that progressed after treatment in this study was 20 Gy/1f, with an effective peripheral biological dose of 60 Gy, which is a low dose compared with previous reports in the literature, and the tumor control was poor. Therefore, for larger lymph nodes or lymph nodes with unclear relationship to surrounding tissues, it is unknown whether increasing the brachytherapy dose can improve the local remission rate while ensuring that normal tissues can tolerate it, and further studies are needed.

In terms of adverse reactions, there were four patients of skin injury: grade II, six patients of grade I, and no grade III or IV skin injury; bone marrow suppression: two patients of leukopenia grade I; no serious complications (e.g., hemorrhage, infection, or other organ damage). Grade III-IV complications occurred in 13% of patients in the study by Nikolaos Tselis et al [7]. In the study by Christos Kolotas et al [8], for skin reactions, 13% grade II reactions and 4% grade III reactions were seen. No carotid bursts or hemorrhages were seen in any of the patients. No late complications occurred in any of the patients. The lesser radioskin injury in the present study compared to the present study may be considered to be related to the small size and location of the recurrent lymph nodes in our patients. In contrast, Lee et al. [15]reported that grade 3–4 radiotherapy adverse reactions occurred in 38% of 21 patients with neck lymph node recurrence who underwent re-course radiotherapy. Wang Juan [12] et al. have suggested that acute cutaneous radiotherapy reactions after particle implantation were re-evaluated: eight patients of grade II, 7 patients of grade I, and two patients of grade 0. No significant serious complications (e.g., spinal cord damage, pharyngeal edema, infection, and bleeding) were identified. As indicated by the above result, in terms of adverse reactions, the insertion of post-implantation radiotherapy is not inferior to particle implantation treatment and is significantly better than re-course radiotherapy.

This study also had some limitations: (1) as a retrospective study, the sample size was small and the patients underwent multiple treatment modalities, such that the sample was heterogeneous to a certain extent; (2) to set the prescribed dose, the size and location of the tumors of the patients were considered, and a more flexible individualized treatment plan was adopted, such causing differences in the prescribed dose (single dose of 20–30 Gy). In brief, ^192^Ir high-dose-rate brachytherapy shows potential advantages as an alternative local treatment for recurrent or residual neck metastatic lymph nodes in the field after external radiotherapy for its recent efficacy (especially for ≤ 3 cm lymph nodes) and low incidence and tolerability of adverse events. In patients with cervical lymphadenopathy who have recurrent head and neck tumors,^192^Ir high-dose-rate brachytherapy, plays an important role in providing palliative care and tumor control [7, 8]. Still, the small number of patients and short follow-up time should be studied in depth.

## Data Availability

The datasets generated and/or analysed during the current study are not publicly available due to limitations of ethical approval involving the patient data and anonymity but are available from the corresponding author on reasonable request.

## Abbreviations

CR: Complete response
PR: Partial response
SD: Stable disease
PD: Progressive disease
RTOG: Radiation Therapy Oncology Group
ECOG: Eastern Cooperative Oncology Group
PGTV: The planning gross tumor volume
PGTVnd: The planning gross target volume of metastatic lymph nod
PTV: Planning target volume
PET-CT: Positron emission tomography-computed tomography
CT: Computed tomography
